# Inhibition of multiple voltage-gated calcium channels may contribute to spinally mediated analgesia by epipregnanolone in a rat model of surgical paw incision

**DOI:** 10.1080/19336950.2018.1564420

**Published:** 2019-01-23

**Authors:** Sonja Lj. Joksimovic, Rebecca R. Donald, Ji-Yong Park, Slobodan M. Todorovic

**Affiliations:** a Department of Anesthesiology, University of Colorado Denver, Aurora, CO, USA; b Department of Anesthesiology, Duke University Medical School, Durham, NC, USA; c Department of Anesthesiology and Pain Medicine, College of Medicine, Korea University, Seoul, Republic of Korea; d Neuroscience Graduate Program, University of Colorado Denver, Aurora, CO, USA

**Keywords:** Neurosteroids, hyperalgesia, calcium, low voltage-activated calcium channels

## Abstract

Voltage-activated calcium channels play an important role in excitability of sensory nociceptive neurons in acute and chronic pain models. We have previously shown that low-voltage-activated calcium channels, or T-type channels (T-channels), increase excitability of sensory neurons after surgical incision in rats. We have also found that endogenous 5β-reduced neuroactive steroid epipregnanolone [(3β,5β)-3-hydroxypregnan-20-one] blocked isolated T-currents in dorsal root ganglion (DRG) cells *in vitro*, and reduced nociceptive behavior *in vivo*, after local intraplantar application into the foot pads of heathy rats and mice. Here, we investigated if epipregnanolone exerts an antinociceptive effect after intrathecal (i.t.) application in healthy rats, as well as an antihyperalgesic effect in a postsurgical pain model. We also studied if this endogenous neurosteroid blocks currents originating from high voltage-activated (HVA) calcium channels in rat sensory neurons. In *in vivo* studies, we found that epipregnanolone alleviated thermal and mechanical nociception in healthy rats after i.t. administration without affecting their sensory-motor abilities. Furthermore, epipregnanolone effectively reduced mechanical hyperalgesia after i.t application in rats after surgery. In subsequent *in vitro* studies, we found that epipregnanolone blocked isolated HVA currents in nociceptive sensory neurons with an IC_50_ of 3.3 μM in a G-protein-dependent fashion. We conclude that neurosteroids that have combined inhibitory effects on T-type and HVA calcium currents may be suitable for development of novel pain therapies during the perioperative period.

## Introduction

Despite the fact that numerous life-saving surgical procedures are performed on a daily basis, treatment of pain arising after surgery continues to be an underestimated problem in patients. One of the reasons is suboptimal clinical pain management, which often leads to the development of chronic pain after surgery [1,2]. Opiate analgesics are commonly prescribed medicines for the treatment of post-operative pain, even though their application is often followed by a plethora of adverse events, complicating post-surgical outcomes and patient recovery [3,4]. Therefore, there is an increased recognition of the need to improve our scientific and clinical knowledge in the field of post-operative pain management and investigate novel treatments for alleviating pain with fewer adverse events.

Several subtypes of voltage-gated calcium channels (VGCCs) are expressed in neurons in the pain pathways, and are crucial not only in shaping action potentials, but also in controlling cellular excitability and synaptic transmission in these cells. On the basis of the membrane potential at which they become activated, VGCCs are subdivided into two major classes: high-voltage-activated (HVA) or sustained currents; and low-voltage-activated (LVA) or transient (T-type) currents [5]. These channels are products of different genes that encode α1 subunits, which form the pores of the VGCCs. The genes can be grouped into families based on α1 subunits: Ca_V_1 (former α1S, α1C, α1D, α1F) encoding L-type channels; Ca_V_2.1 (α1A) encoding P/Q-type channels; Ca_V_2.2 (α1B) encoding N-type channels; and Ca_V_2.3 (α1E) encoding R-type channels, which activate at more negative potentials than other HVA channels.

The supportive role of T-channels in pain pathways has been relatively well established. Consistent with this notion, blocking peripheral T-channels leads to a very potent antinociceptive effect in models of somatic [6–9] and visceral pain [10]. Also along these lines, we reported recently that upregulation of the Ca_V_3.2 isoform of T-channels in DRG sensory neurons supports the development and maintenance of hyperalgesia after surgical incision [11]. Several other studies have investigated the role of HVA calcium channels in postsurgical pain models. Wang and colleagues applied ziconotide, an N-type calcium channel blocker, intrathecally after skin incision, and this alleviated thermal hyperalgesia in a dose-dependent manner [12]. In addition, Field et al. [13] demonstrated preventative application of gabapentin, a ligand for the α_2_δ ancillary subunits of VGCCs, relieved postsurgical thermal hyperalgesia and tactile allodynia in incised rats.

**Figure 1.**
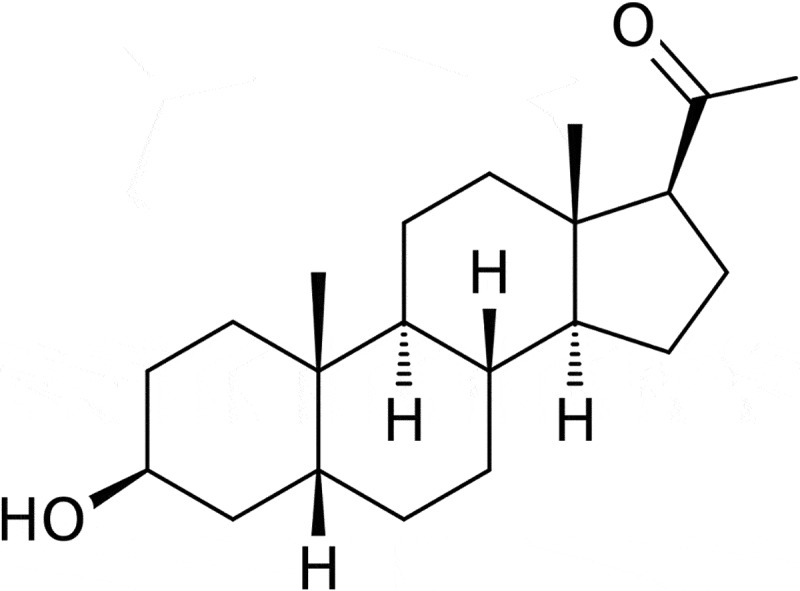
The chemical structure of epipregnanolone.

An increasing body of evidence suggests that endogenous neurosteroids and their synthetic analogues have significant potential as analgesics [14]. Recent studies have confirmed the production of neurosteroids in the spinal cord, a pivotal part of the central nervous system for transmission of information regarding painful stimuli from the periphery [15–17]. Neurosteroids exert their various effects on the sensory transmission by modulating numerous receptor-operated systems (GABA_A_, NMDA, nuclear receptors) and various VGCCs [18–21]. Several studies have implicated endogenous neurosteroids, such as pregnane and its derivatives, in the prevention of allodynia in neuropathic pain, as well as mechanical and thermal hyperalgesia in an inflammatory pain model in rats [17,22,23]. Our previous study showed that 5α-reduced neurosteroids exert their analgesic effects mostly by interacting with GABA_A_ receptors and T-channels in peripheral nociceptive neurons [24]. We have also shown that in rat and mouse dorsal root ganglia (DRG) sensory neurons, the endogenous 5β-reduced neurosteroid epipregnanolone [(3β,5β)-3-hydroxypregnan-20-one] (Figure 1) exerts potent analgesia in healthy rats and mice as a result of inhibition of the Ca_V_3.2 isoform of T-channels, without having an effect on GABA_A_-mediated currents [25]. We have also found that that the ability of synthetic 5β-reduced neurosteroids to produce an antinociceptive effect in healthy rats correlates well with their potency in blocking Ca_V_3.2 channels in rat DRG sensory neurons [26].

In this study, we investigated analgesic properties of epipregnanolone on surgically induced hyperalgesia using a plantar incision of the hind paw in rats [27], which is a rodent model considered to be similar in context to the underlying mechanisms and the development of human post-operative pain [28]. Given the importance of multiple subtypes of VGCCs in pain transmission, this study also examined the effects of epipregnanolone on isolated HVA calcium currents in nociceptive sensory neurons in the DRG.

## Materials and methods

### Animals

All *in vivo* experiments were performed with young female Sprague-Dawley rats (P25-P70; Envigo, Indianapolis, IN, USA). Animals were housed two per cage and maintained on a 12 h light-dark cycle with access to food and water *ad libitum*. Experimental protocols were in accordance with the Guide for the Care and Use of Laboratory Animals (Institute of Laboratory Animal Resources, 1996), and were approved by the Animal Care and Use Committee of the University of Colorado Anschutz Medical Campus and the University of Virginia.

### Drugs

Epipregnanolone [(3β,5β)-3-hydroxypregnan-20-one] is an endogenously produced neurosteroid that we obtained from Steraloids, Inc. (Newport, RI). For intrathecal (i.t.) injections, the compound was dissolved in a solution of 15% 2-Hydroxypropyl-β-cyclodextrin in pH-balanced saline (pH = 7.4 to avoid tissue irritation). 2-Hydroxypropyl-β-cyclodextrin (45% solution) was purchased from Santa Cruz Biotechnology (Santa Cruz, CA).

#### Incisional pain model

In order to study the antihyperalgesic effect of epipregnanolone after surgery, we used a skin and muscle incision for the induction of post-surgical pain [27]. Animals were anesthetized with isoflurane (2.5% for induction and for maintenance) and the plantar surface of the right paw was incised longitudinally with a No.11 surgical blade. The underlying plantaris muscle was elevated and also incised longitudinally, after which the skin was sutured with 5–0 nylon suture using an FS-2 needle. Animals were allowed to recover in cages, and all experiments were initiated as early as 24 h post-incision.

#### Intrathecal injections

After briefly anesthetizing animals with isoflurane (2.5% for induction in an induction chamber, and 2.5% for maintenance), the back of each animal was shaved to expose the injection site in the L_4_-L_6_ region of the spinal column. A 28 G needle was used for the acute i.t. injection. After inserting the needle into the L_4_-L_6_ lumbar region, the experimental compound or vehicle was delivered in a volume of 50 µl, and the animal was left to recover in a cage before initiating experiments.

#### Thermal nociception testing

For assessment of thermal (heat) nociception threshold, experiments were performed as previously described [11]. In brief, after 15 minutes of acclimation, a radiant heat source is positioned directly underneath a plantar surface of the hind paws to deliver a thermal stimulus and an automatic timer begins. When the animal withdrew the paw, the automatic timer shut off, therefore measuring the animal’s paw withdrawal latency (PWL). To prevent thermal injury, the light beam was automatically discontinued at 20 s, if the rat failed to withdraw its paw. For this study, each paw was tested three times and the average value of the PWLs was used in further analysis.

#### Mechanical sensitivity

In order to determine mechanical sensitivity in rats, we used the electronic Von Frey apparatus (Ugo Basile, Varese, Italy) as previously described [11]. In brief, after habituation of the animals in plastic enclosures, a rigid metal probe was applied to the plantar surface of the paw through the mesh floor of the stand, and constant force, in a range of 0–50 g, was applied to the mid-plantar area of the paw. As soon as the exerted pressure of the punctate stimulus reached the maximum force that the animal can endure, immediate brisk paw withdrawal was noticeable, and the force in grams was displayed on the apparatus representing a threshold for paw withdrawal response (PWR). In this study, each paw was tested three times and the average value of threshold PWRs was used in further analysis. Any other voluntary movement of the animal was not considered as a response.

#### Assessment of sensorimotor abilities

To eliminate the possible effect of the highest applied dose of the tested drug on sensorimotor abilities [29], we tested unoperated rats before and after an i.t. injection of 300 µM epipregnanolone. Animals were tested on four different sensorimotor tasks: ledge, walking initiation, elevated platform, and inclined plane. Ledge experiments measured the number of seconds the rat remained on a 3 cm-wide plank. Means for each animal were calculated over two trials, with a maximum of 60 seconds per trial. Walking initiation experiments were conducted by placing a rat in the center of a 50 cm x 50 cm square made with pieces of tape on a desk. Time was measured as the number of seconds necessary for the rat to move all four paws off the square. For elevated platform experiments, a rat was placed on a platform (7.6 x 15.2 cm) positioned 61 cm above the ground. Time was measured by the number of seconds the animal could remain on the platform. A mean value was calculated from two trials with a maximum of 120 seconds (test cut-off value). For inclined plane experiments, the rat was placed in the middle of a wire mesh screen (8 squares in 10 cm) tilted at a 60° angle with head facing down. Time was measured by the number of seconds the rat could remain on the inclined screen before falling off. The cut-off value was 120 seconds, which was assumed to be the maximum time an animal could remain on the inclined screen.

#### Tissue preparation for electrophysiological recordings

Dissociated cell bodies from neurons of the dorsal root ganglion (DRG) were harvested from adolescent rats of both sexes and prepared as we previously described [11]. Briefly, after anesthetizing the animals with 5% isoflurane, and decapitating with a guillotine, DRGs were removed and immediately placed in ice-cold Tyrode’s solution, containing 140 mM NaCl, 4 mM KCl, 2 mM MgCl2, 10 mM glucose, and 10 mM Hepes, adjusted to pH 7.4 with NaOH. After harvesting, the tissue was transferred into Tyrode’s solution containing collagenase H (Sigma-Aldrich) and dispase II (Roche) and incubated for 50 min at 35°C. Dissociated single neuronal cell bodies were obtained by trituration in Tyrode’s solution at room temperature through fire-polished pipettes of progressively reduced sized tips. After trituration, cells were plated onto uncoated glass coverslips, placed in a culture dish, and perfused with external solution. All *in vitro* experiments were done at room temperature. Only small-sized DRG cells with a soma diameter < 30 μm were used in recordings. All recordings used in this study were obtained from a total of 51 DRG cells from 28 rats with an average soma diameter of 22.9 ± 4.1 μm.

#### Electrophysiological methods

Recordings were made with standard whole-cell, voltage-clamp techniques previously described [11]. Pipette resistances were 2 to 5 MΩ. Reported series resistance and capacitance values were taken from the reading of the amplifier. Typically cells were held at −50 mV and depolarized to −10 mV every 20 seconds to evoke inward currents. Data were analyzed using Clampfit 10.4 (Axon Instruments, Foster City, CA) and Origin 7 (Microcal Software, Northhampton, MA). Currents were filtered at 5 kHz. All experiments were done at room temperature (20–23°C). In most experiments, leakage subtraction was used with a P/5 protocol for on-line leakage subtraction.

The external solution for electrophysiology recordings of Ca^2+^ currents in DRG cells contained: 160 mM TEA-Cl, 10 mM HEPES, 5 to 10 mM BaCl2, adjusted to pH 7.4 with TEA-OH; osmolarity, 316 mOsM. Cells were generally maintained in a Tyrode’s solution until seal formation, at which time the bath solution was switched to the Ba^2+^ saline. The internal solution consisted of 110 mM Cs-methane sulfonate, 14 mM phosphocreatine, 10 mM HEPES, 9 mM EGTA, 5 mM Mg-ATP, and 0.3 mM tris-GTP, pH adjusted to 7.15 to 7.20 with CsOH (standard osmolarity, 300 mOsM). In some experiments, GTP in the internal solution was preplaced with its inactive analogs 100 μM GTP-γ-S or 2 mM GDP-β-S in order to investigate the role of G-proteins in inhibition of HVA calcium currents. Another method to interrogate the role of G-proteins involves strong, brief depolarization (e.g. +100 mV), which would be expected to relieve G-protein-mediated inhibition of HVA calcium currents [30]. We have not performed such experiments with strong depolarizing prepulse relief in our acute preparation of DRG cells because of difficulties in keeping stable whole-cell recordings following extremely positive membrane potentials.

#### Data analysis and statistics

Each data point from behavioral experiments was expressed as mean ± SEM. The differences in effects between the treatment and the vehicle groups was performed using two-way repeated measures analysis of variance (RM-ANOVA) followed by Sidak’s post-hoc test, and paired t-test. Signiﬁcant differences between group means are indicated when p < 0.05. GraphPad Prism 7 was used for all statistical analyses. Origin 7 was used to calculate ED_50_.

For the concentration-response curve of epipregnanolone inhibition of calcium currents, all points are averages of multiple determinations obtained from at least 3 different cells. All concentrations were applied to the cells until an apparent steady state effect was achieved. On all plots, vertical bars indicate standard errors. Mean values on concentration-response curves were fit to the Hill-Langmuir function: PB([Epipregnanolone]) = PB_max_/[1 + (IC_50_/[Epipregnanolone])*n*_H_], where PB_max_ is the maximal percent block of peak current, the IC_50_ is the concentration that produces 50% of maximal inhibition, and *n* is the apparent Hill coefficient for blockade. Fitted values are typically reported with 95% linear confidence limits.

## Results

### Antinociceptive effects of intrathecally administered epipregnanolone on thermal and mechanical nociception in healthy rats

**Figure 2.**
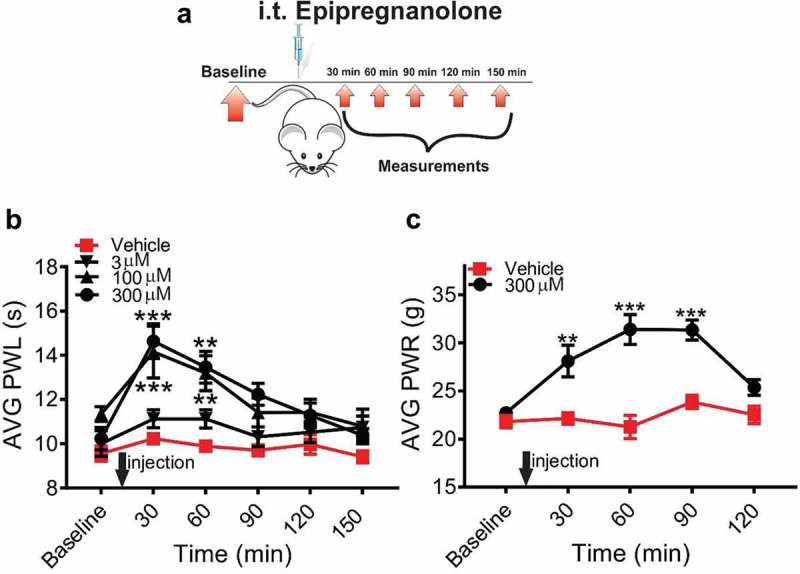
Intrathecal application of epipregnanolone reduces thermal and mechanical nociception in healthy rats. (a). Schematic representation of experimental protocol. Baseline measurements of responses to heat and mechanical stimuli were measured over two days before injection. Following an intrathecal (i. t.) injection of epipregnanolone or vehicle, thermal and mechanical nociception were assessed over time. (b). Graph of paw withdrawal latency (PWL) following i.t. injection of vehicle or epipregnanolone at 3, 100, and 300 µM, each applied in a volume of 50 µl, showing epipregnanolone alleviates thermal nociception in a dose dependent manner (n = 9–12 animals per group; two-way RM ANOVA with Sidak’s post-hoc test; interaction: F(12,144) = 2.498, p = 0.0053; treatment: F(3,36) = 7.123, p < 0.001; at 30 min: vehicle vs 100 µM: *** p < 0.001, vehicle vs 300 µM: *** p < 0.0001; at 60 min: vehicle vs 100 µM: ** p = 0.0064; vehicle vs 300 µM: ** p = 0.0038). (c). Graph of paw withdrawal response (PWR) following i.t. injection of vehicle or 300 µM epipregnanolone, each applied in a volume of 50 µl, showing the antinociceptive effect of epipregnanolone on mechanical nociception over time (n = 5–7 animals per group; two-way RM-ANOVA with Sidak’s post-hoc test; interaction: F(3,30) = 3.491; p = 0.0277; treatment: F(1,10) = 43.43, p < 0.0001; at 30 min: vehicle vs 300 µM: ** p = 0.0054; at 60 min: vehicle vs 300 µM: *** p < 0.001; at 90 min: vehicle vs 300 µM:*** p < 0.001). Each data point represents the mean ± SEM.

Our previous study demonstrated that when injected into peripheral receptive fields of a rat’s paw, epipregnanolone exerted a potent antinociceptive effect on a thermal nociceptive stimulus [25]. Here, we examined if local intrathecal (i.t) application of epipregnanolone could alleviate thermal and mechanical nociception in healthy rats. Animals were injected i.t. with epipregnanolone and nociceptive measurements began 30 minutes post-injection (Figure 2(a)). Indeed, epipregnanolone exerted a substantial dose-dependent antinociceptive effect to a thermal stimulus during 150 minutes post i.t. injection as indicated by a transient increase in the thermal PWLs up to 45% when compared to pre-injection baselines. The peak effect was observed 30 minutes after injection (Figure 2(b)). The compound was tested at three concentrations (3, 100 and 300 µM) each applied in a volume of 50 µl. While the concentrations of 100 and 300 µM, each applied in a volume of 50 µl, exerted a prominent antinociceptive effect, the lowest concentration of 3 µM, in a volume of 50 µl, did not offer any significant alleviation of nociceptive behavior in thermal nociceptive testing. In order to investigate the effects of epipregnanolone on mechanical nociception, we used the highest effective concentration of epipregnanolone from the previous experiments with thermal nociception (Figure 2(b)). As expected, 300 µM epipregnanolone, applied in a volume of 50 µl, markedly reduced responsiveness to mechanical stimulus after i.t. application in healthy rats (Figure 2(c)), as indicated by an increase in the average PWLRs of about 30% which persisted for 90 minutes. The effect of epipregnanolone on mechanical nociception was longer lasting than for thermal nociception (compare Figures 2(b, c)).

### Effect of epipregnanolone on sensory-motor abilities of the injected healthy rats

**Figure 3.**
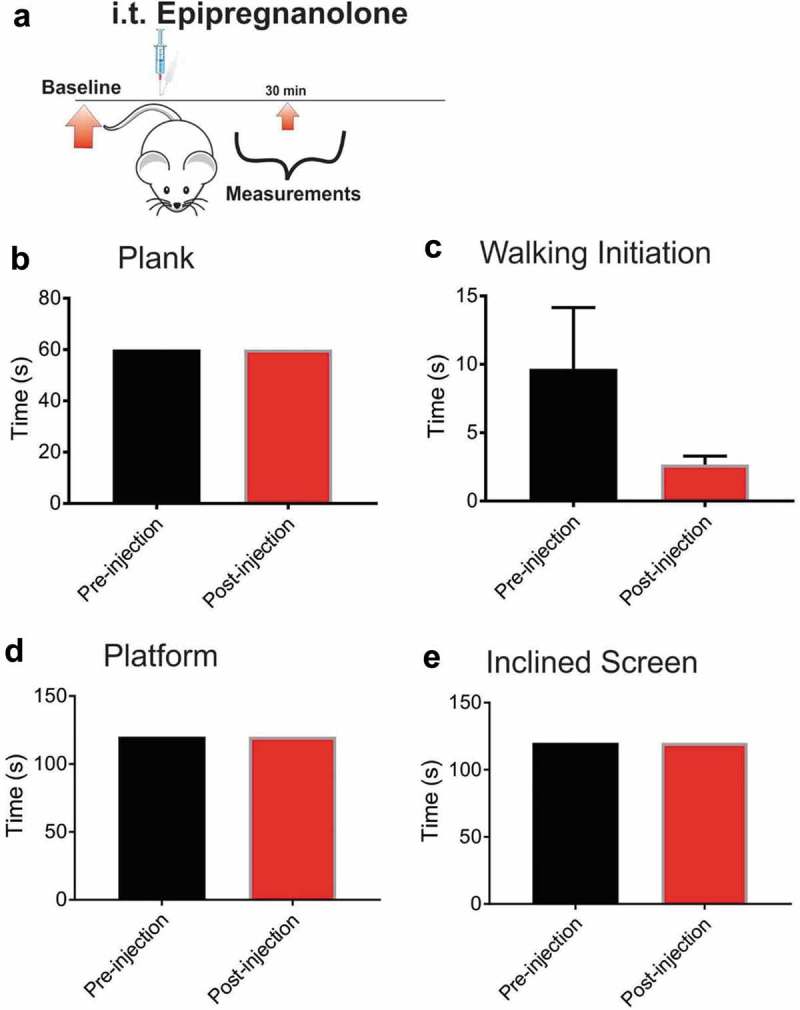
Intrathecal application of epipregnanolone does not impair sensory-motor abilities of rats. (a). Schematic representation of experimental protocol for baseline measures of sensorimotor abilities prior to injection, and assessment performed after an intrathecal injection (i.t.) of 50 µl of 300 µM epipregnanolone. (b). Bar graph demonstrates time rats remained on the plank before and after an i.t. injection of 50 µl of 300 µM epipregnanolone (n = 6 rats for each group). (c). Bar graph of walking initiation demonstrates time required for rats to place all four paws outside the labeled square before and after i.t. injection of 50 µl of 300 µM epipregnanolone (n = 6 rats for each group, paired t-test, p = 0.21). (d). Bar graph demonstrates time rats remained on elevated platform before and after i.t. injection of 50 µl of 300 µM epipregnanolone (n = 6 rats for each group) (e). Bar graph demonstrates time rats remained on inclined screen before and after i.t. injection of 50 µl of 300 µM epipregnanolone (n = 6 rats for each group).

Responsiveness to painful stimuli could be reduced if injection of epipregnanolone interferes with the motor capabilities of animals. Therefore, we tested the sensorimotor abilities of healthy rats before and 30 minutes after neurosteroid injection (Figure 3(a)). Sensorimotor performance on the plank (Figure 3(b)), walking initiation (Figure 3(c)), elevated platform (Figure 3(d)) or inclined screen (Figure 3(e)) did not differ significantly before or after an i.t. injection of 300 µM epipregnanolone, applied in a volume of 50 µl. Although the time needed to walk out of the designated square on the walking initiation task was shorter post-injection compared to pre-injection, the difference in time was not statistically significant. Hence, we concluded that any decrease in response to painful stimuli after injections of epipregnanolone would not be a consequence of motor impairment.

### Effects of single intrathecal application of epipregnanolone on post-incision hyperalgesia in rats

**Figure 4.**
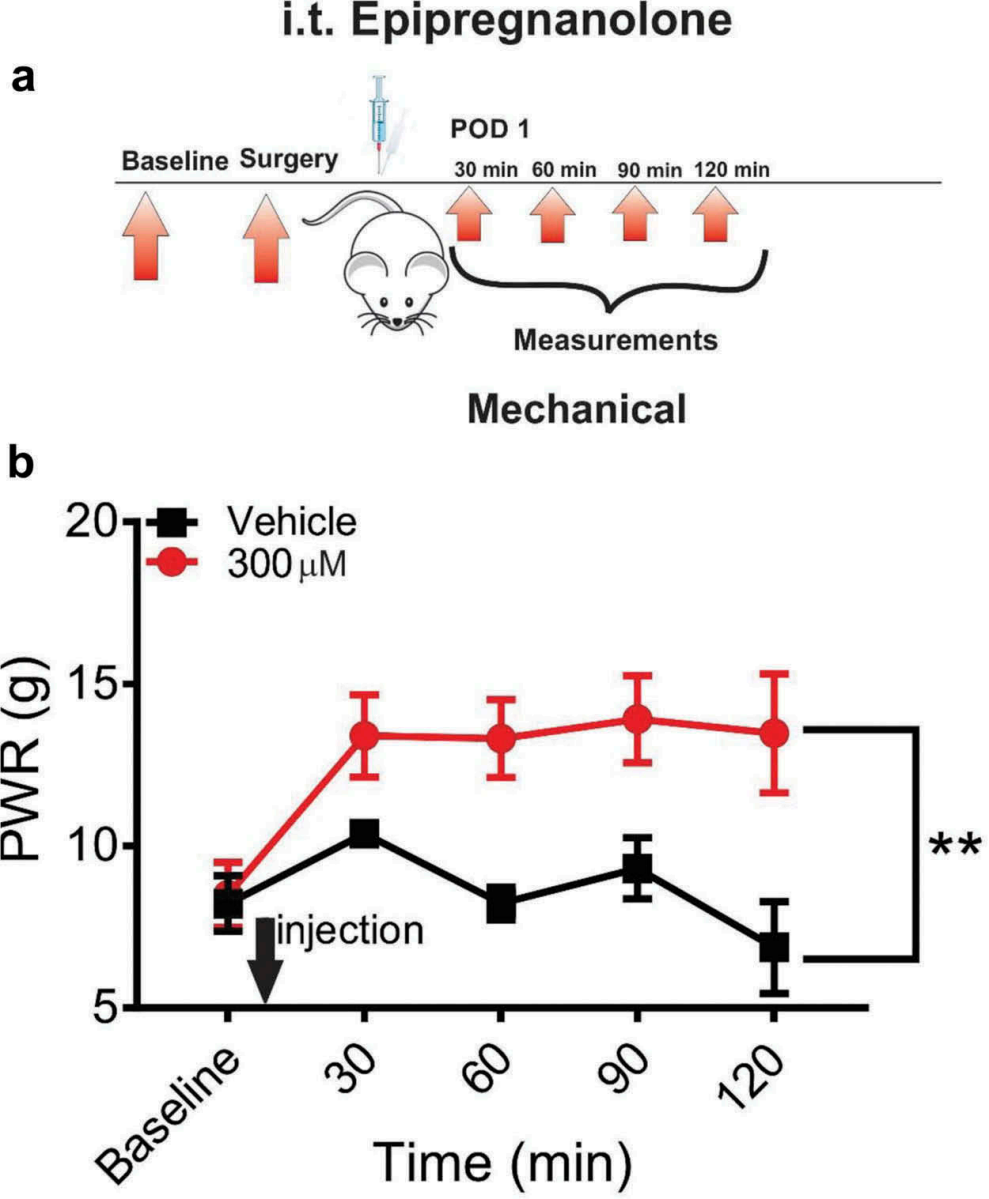
Single intrathecal application of epipregnanolone reduces mechanical nociception in incised rats. (a). Schematic representation of protocol. Baseline measurements of paw withdrawal response (PWR) following mechanical stimulus determined two days before the incision, and assessment post-injection in surgically incised rats. Intrathecal (i.t.) injections of vehicle or 300µM epipregnanolone, each applied in a volume of 50 µl, were performed 24 h post-incision followed by assessment of mechanical hyperalgesia at 30, 60, 90 and 120 minutes post-injection. (b). Graph of time-course for PWR following i.t. injection of vehicle or 300µM epipregnanolone, each applied in a volume of 50 µl, showing antihyperalgesic effect of epipregnanolone on mechanical nociception (n = 4–7 rats per group, two-way RM ANOVA, interaction: p = 0.61; treatment: F(1,9) = 15.01, ** p = 0.0038). Each data point represents the mean ± SEM.

To determine if epipregnanolone alleviates post-surgical hypersensitivity, we injected rats i.t. with either 50 µl of 300 µM epipregnanolone or vehicle at the time point of 24 h after a surgical incision and measured the response to a mechanical stimulus over a period of 120 minutes post-injection (Figure 4(a)). We chose to perform our experiments 24 h after surgery, because previous study had shown that hyperalgesia is fully developed at 24 and 48 h after surgery [11]. Our experiments in healthy rats showed that an injection of 50 µl of 300 µM epipregnanolone exerted the most prominent antinociceptive effect (Figure 2(c)). Hence, we used this concentration in our experiments with post-surgical pain model. We found that a single i.t. injection of 50 µl of 300 µM epipregnanolone effectively alleviated mechanical hyperalgesia as evidenced by a lasting increase in PWRs of about 2-fold when compared to the pre-injection baselines (Figure 4(b)). Importantly, the antihyperalgesic response to a mechanical punctate stimulus was significantly greater than vehicle injected rats over the entire period of 120 minutes post-injection (p = 0.0038).

### Epipregnanolone inhibits high-voltage-activated (HVA) calcium currents in rat sensory neurons in a G-protein-dependent fashion

The identity of different HVA current components in DRG neurons depends to a large extent on the diameter of the underlying cells [31]. For example, small-diameter neurons typically are enriched in L- and N-type HVA currents [31]. In contrast, larger DRG neurons express a larger portion of non-L, non-N currents, which has been reported to be of the P-type [32], Q-type [33], and a component unblocked by any known blocker [33], presumably R-type. Furthermore, it is generally accepted that that the majority of small size DRG neurons are nociceptors. Therefore, for examination of the effects of epipregnanolone on HVA currents in DRG neurons, we selected only smaller-diameter cells.

**Figure 5.**
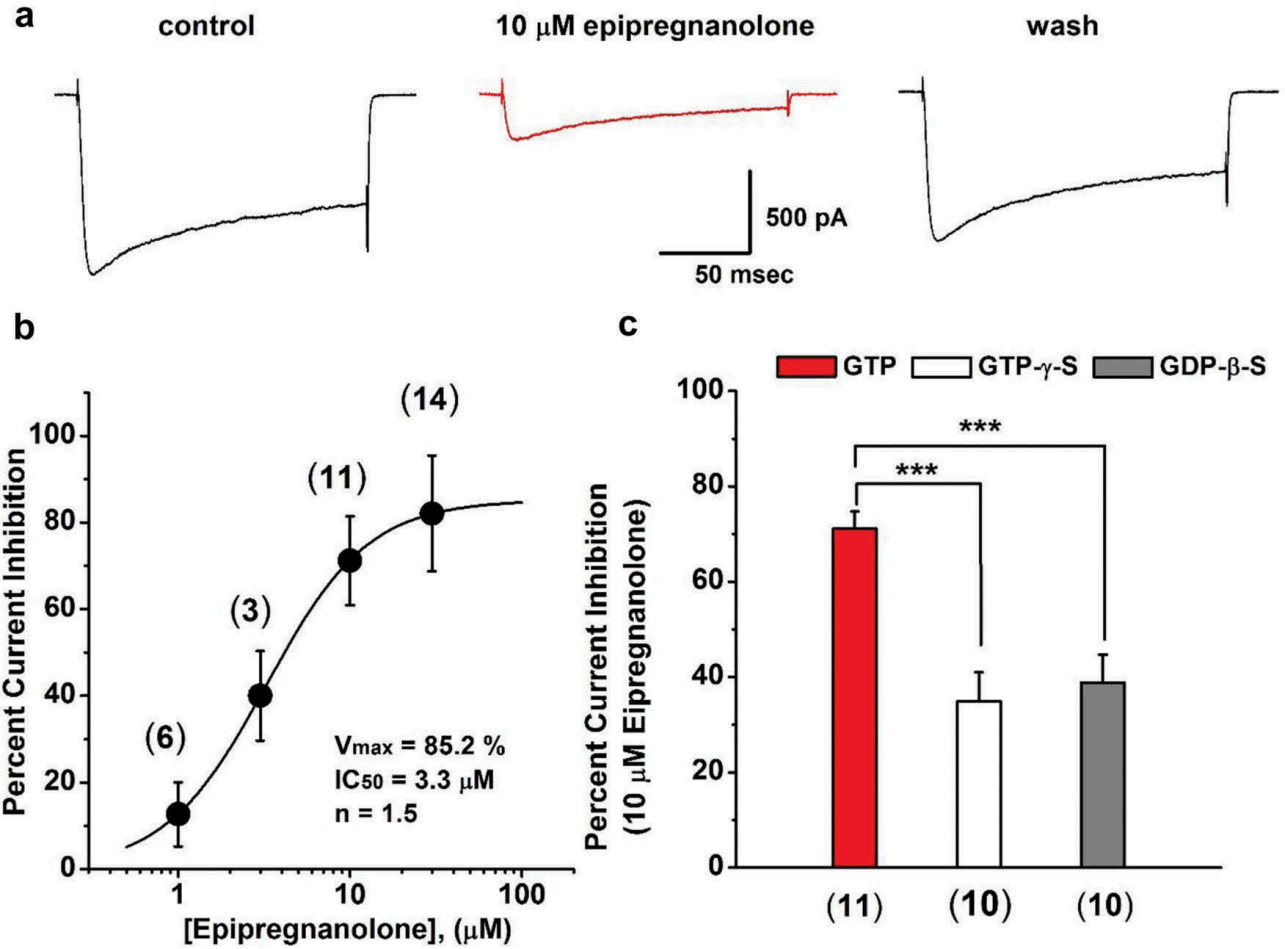
Epipregnanolone inhibits high-voltage-activated (HVA) calcium currents in rat sensory neurons in a G-protein-dependent fashion. (a). Representative traces of evoked HVA currents (V*h* −50 mV, V*t* −10 mV) recorded from dissociated DRG neurons of healthy animals; the traces were recorded before (control), 4 minutes after the application of 10 µM epipregnanolone, and after a 4 minute washout of the neurosteroid (wash). (b). The concentration-response curve shows the percentage of current inhibition achieved after application of a range of different concentrations of epipregnanolone. Each point is the average of at least three different cells (total n = 34 cells, the exact number of cells per group is presented on the graph) ± SEM; solid line indicates best fit using unrestricted concentration-response equation, giving 85% for maximal current inhibition (V_max_), IC_50_ of 3.3 µM, and Hill coefficient 1.5. (c). HVA current inhibition of dissociated DRG. Red bar indicates inhibition with 10 μM epipregnanolone in the presence of GTP in the internal solution (red bar) is approximately 70% (n = 11 cells). Inhibition of HVA currents by epipregnanolone is reduced in the presence of GTP-γ-S (white bar, n = 10 cells, p < 0.001, Unpaired t-test) or GTP-β-S (gray bar, n = 10 cells, p < 0.001, Mann-Whitney test), as compared to the red bar.

In order to investigate if antihyperalgesic effects of epipregnanolone might, at least in part, be due to its inhibitory effects on HVA calcium channels, we compared the amplitudes of evoked calcium currents in the same DRG neurons in the control conditions before drug applications, and in the presence of 10 µM epipregnanolone. Indeed, we found that 10 μM epipregnanolone effectively reduced peak HVA currents (Figure 5(a)) compared to control baseline currents in the same cells. Washout of epipregnanolone resulted in a return of HVA currents close to pretreatment levels (Figure 5(a)), demonstrating the block was reversible. Furthermore, epipregnanolone inhibited HVA currents in a concentration-dependent manner, with an IC_50_ of 3.3 µM; an 85% maximal block indicated nearly complete inhibition (Figure 5(b)). Next, in order to study the mechanisms of the interaction between HVA calcium channels and epipregnanolone, we examined whether G-proteins could be involved in the inhibition of HVA currents in DRG neurons by adding known activators and inhibitors of G proteins to the internal recording solution (Figure 5(c)). Under control conditions of recording the effects of epipregnanolone in the presence of GTP, approximately 70% inhibition of HVA currents occurred in DRG neurons (Figure 5(c), red bar). In the next set of experiments activation of G-proteins was achieved by adding 100 µM GTP-γ-S to the recording solution; GTP-γ-S activates G-proteins persistently and prevents subsequent effects of G-protein-dependent agonists. A significant reduction of the epipregnanolone-induced inhibition of HVA (p < .001) occurred in the presence of GTP-γ-S (Figure 5(c), white bar). To validate this finding in another set of independent experiments, inhibition of G-protein–mediated signaling was next achieved by introducing 2 mM GDP-β-S, an antagonist of G-protein activation into the recording pipette [34]. Similar to GTP-γ-S, we found that GDP-β-S significantly diminished epipregnanolone-induced inhibition of HVA currents in DRG cells (p < .001) to approximately 50% of control response (Figure 5(c), gray bar).

These results are consistent with a notion that HVA current reduction occurring after epipregnanolone application is most likely G-protein dependent.

## Discussion

To our knowledge, this is the first study investigating the analgesic potential of an endogenous neurosteroid in an acute somatic postsurgical pain model. The discovery of endogenous neurosteroids present in the spinal cord, as well as the enzymes involved in steroidogenesis at both spinal and supraspinal levels [35], is strongly suggestive of their involvement in modulation of pain transmission [16,36]. It is already established that endogenously produced 5α-reduced derivatives of progesterone, such as allopregnanolone and dihydroprogesterone, exert antihyperalgesic effects in neuropathic and inflammatory pain models [17,24,37–41]. Epipregnanolone belongs to the group of 5β-reduced endogenous neurosteroids derived from progesterone by the actions of 5β-reductase and 3β-hydroxysteroid dehydrogenase. We have previously shown that local intraplantar application of epipregnanolone reduced both thermal and mechanical nociception in the wild type mice, but was ineffective in Ca_V_3.2 knock-out mice [25].

Our *in vivo* methods demonstrated that epipregnanolone exerts an antinociceptive effect after i.t. application in healthy rats and an effective antihyperalgesic effect in a postsurgical pain model. An i.t. application of epipregnanolone was employed in our studies because it represents a common route of administration of analgesics in clinical practice [42]. Indeed, an i.t. application of epipregnanolone alleviated thermal nociception in healthy rats in a dose-dependent manner. Importantly, we demonstrated that an i.t. application of 50 µl of 300 µM epipregnanolone, the highest effective concentration, did not interfere with sensorimotor function of healthy rats. Additionally, the same concentration of epipregnanolone applied intrathecally in healthy rats also reduced mechanical sensitivity. Finally, in surgically incised rats, an i.t. injection of 50 µl of 300 µM epipregnanolone on postsurgical day 1 effectively attenuated mechanical hyperalgesia.

Previous studies have reported that non-NMDA [43,44] and opioid receptors [45] are involved in the maintenance of incisional pain, however the exact mechanisms and mediators involved in the development of incisional pain have not been determined. It is well established that VGCCs (particularly T-type and N-type) contribute to the transmission of nociceptive signals in primary afferent fibers [46,47]. In addition, we know the Ca_V_3.2 T-channel isoform is present on the cell bodies of peripheral nociceptive neurons in the DRG [48,49], and on the presynaptic terminals in laminae I and II of the dorsal horn of the spinal cord, thus facilitating excitatory neurotransmission [50]. We recently demonstrated an increase in current density of T-channels on the cell bodies of DRG sensory neurons in a surgical incision pain model [11]. In addition, we previously found that epipregnanolone exerts antinociceptive effects in healthy rats, in part by reversibly blocking T-channels in peripheral DRG neurons with an IC_50_ of 3 μM [25]. In the same study, we reported that epipregnanolone did not have any effect on sodium, potassium and gamma-aminobutyric acid (GABA)-gated ionic currents in DRG neurons. We continue here by showing that epipregnanolone inhibits HVA calcium currents in nociceptive DRG neurons with similar potency as reported for T-currents. Collectively, these findings suggest that antihyperalgesic effect of epipregnanolone in the postsurgical pain model may be mediated in concert *via* inhibition of T-channels and HVA channels in nociceptors.

It is also important to speculate on the nature of HVA calcium current in DRG neurons that are inhibited by epipregnanolone. It is clear that the transduction and processing of pain signals are dependent on, and modulated by, various ion channels and receptors [51]. Therefore, besides T-channels, N-type (Ca_v_2.2) VGCCs emerge as possible targets involved in modulation of pain perception and transmission of pain signals. The N-type channel consists of α_1B_- (Ca_V_2.2), β-, and α_2_δ-subunits forming a hetero-oligomer [52]. They are expressed only in neuronal tissue, with particularly high levels of expression in the lamina I and II of the dorsal horn, which represent a main pain-processing region of the spinal cord. It has been reported that blocking N-type channels prevents the release of neuropeptides from primary afferents in the dorsal horn such as substance P [53]. Furthermore, inhibition of trafficking of the Ca_V_2.2 channel reduces inflammatory and postsurgical pain [54]. Lastly, mutant mice lacking the gene that encodes the N-type channel exhibit less sensitivity in neuropathic and inflammatory pain models, when compared with wild type mice [55,56].

Field and colleagues [13] demonstrated that gabapentin administered subcutaneously reduced thermal and mechanical hyperalgesia in a postsurgical pain model in rats. Gabapentin exerts its effect likely by binding to the α_2_δ-subunit of VGCCs, therefore these findings also suggest a possible involvement of HVA calcium channels in the development of postsurgical pain. Next, Wang and colleagues [12] reported that an i.t. bolus injection of ziconotide, an N-type calcium channel blocker, applied after surgery, significantly reduced mechanical and thermal hyperalgesia in incised paws. Furthermore, in the same study, a preemptive i.t. injection of ziconotide reduced both thermal hyperalgesia and mechanical allodynia post-incision. Importantly, clinical trials demonstrated that i.t. ziconotide significantly relieved postsurgical pain in patients, and also reduced morphine consumption [57]. These previous studies strongly implicate N-type HVA calcium channels in the development of postsurgical pain, which prompted us to investigate whether epipregnanolone could block HVA currents in putative nociceptive DRG neurons.

Our *in vitro* studies showed epipregnanolone reduced the amplitudes of HVA currents (mainly N-type and L-type) in putative nociceptors in a concentration-dependent manner. We also demonstrated that GTP-γ-S and GDP-β-S diminished the ability of 10 µM epipregnanolone to block HVA currents, indicating the reduction in HVA currents was G-protein dependent. Other neurosteroids have been reported to produce inhibitory effects on HVA currents in hippocampal neurons [58], as well as acutely dissociated DRG cells [59]. Importantly, by effectively preventing G-protein coupled modulation, GDP-β-S and GTP-γ-S have been shown to indirectly modulate N-type, but not L-type HVA currents in small DRG cells [59]. Furthermore, previous studies have firmly established the role of G-protein regulation of N-type calcium currents in nociceptive DRG neurons [60–62]. Our data strongly suggest that G-protein-dependent inhibition of HVA currents in small DRG neurons is due to epipregnanolone inhibition of N-type currents. L-type calcium channels have also been implicated in nociception. Although pharmacological studies have suggested analgesic potency of traditional L-type blockers [63–65], the role of L-type VGCCs in post-surgical hyperalgesia was not well studied. It has been demonstrated that systemically applied blockers of L-type calcium channels potentiate antinociceptive effect of morphine and other opiate derivatives [66,67]. However, intrathecal application of verapamil, a potent L-type calcium channel blocker, did not show antinociceptive effect in the tail-flick test in rats [68]. Future studies are necessary to assess the involvement of L-type VGCCs in incisional pain development and spinally mediated analgesia post surgery.

Our findings demonstrate that epipregnanolone, an endogenous neurosteroid, can effectively reduce postsurgical hyperalgesia in rats, most likely by targeting the Ca_V_3.2 isoform of T-type calcium channels and N-type HVA calcium channels in nociceptors. Furthermore, our data strongly suggest that the effect of epipregnanolone on HVA calcium channels is, at least in part, mediated *via* G-protein dependent signaling pathways. These data show that neurosteroids that have combined inhibitory effects on T-type and HVA calcium currents may be suitable for development of novel pain therapies in the perioperative period.
